# PAX7 Balances the Cell Cycle Progression via Regulating Expression of *Dnmt3b* and *Apobec2* in Differentiating PSCs

**DOI:** 10.3390/cells10092205

**Published:** 2021-08-26

**Authors:** Anita Florkowska, Igor Meszka, Joanna Nowacka, Monika Granica, Zuzanna Jablonska, Magdalena Zawada, Lukasz Truszkowski, Maria A. Ciemerych, Iwona Grabowska

**Affiliations:** Department of Cytology, Institute of Developmental Biology and Biomedical Sciences, Faculty of Biology, University of Warsaw, Miecznikowa 1, 02-096 Warsaw, Poland; a.florkowska@biol.uw.edu.pl (A.F.); Igormesz@gmail.com (I.M.); nowackaj010@gmail.com (J.N.); mnk.granica@gmail.com (M.G.); zuzanna.w.jablonska@gmail.com (Z.J.); m.zawada@student.uw.edu.pl (M.Z.); lukasz.t@uni-muenster.de (L.T.); ciemerych@biol.uw.edu.pl (M.A.C.)

**Keywords:** mouse, ESCs, iPSCs, stem cells, differentiation, myogenesis, cell cycle, skeletal muscle, teratoma, Pax7, 5azaC

## Abstract

PAX7 transcription factor plays a crucial role in embryonic myogenesis and in adult muscles in which it secures proper function of satellite cells, including regulation of their self renewal. PAX7 downregulation is necessary for the myogenic differentiation of satellite cells induced after muscle damage, what is prerequisite step for regeneration. Using differentiating pluripotent stem cells we documented that the absence of functional PAX7 facilitates proliferation. Such action is executed by the modulation of the expression of two proteins involved in the DNA methylation, i.e., *Dnmt3b* and *Apobec2*. Increase in *Dnmt3b* expression led to the downregulation of the CDK inhibitors and facilitated cell cycle progression. Changes in *Apobec2* expression, on the other hand, differently impacted proliferation/differentiation balance, depending on the experimental model used.

## 1. Introduction

Pluripotent stem cells (PSCs), such as embryonic stem cells (ESCs) or induced pluripotent stem cells (iPSCs), are capable of self-renewal when cultured under appropriate conditions. When triggered with appropriate stimuli they differentiate into any given tissue. Currently available in vitro differentiation assays allow to generate various cell types that can be than used in research and possibly also in therapies. For many cell types the protocols are straightforward and easily repeatable. For other ones, multiple approaches have been tested not necessarily leading to designing the perfect differentiation method. Among the tissues in which development and function are extensively analyzed using PSCs are skeletal muscles. The protocols allowing derivation of myogenic progenitors from PSCs cover various approaches involving overexpression of selected factors, such as PAX3 or PAX7 (e.g., [1,2,3]), using DNA demethylating agents, such as 5-azacitidine (5azaC) (e.g., [4,5,6]), as well as culture under precisely defined conditions (e.g., [7,8], for review see [9]).

Many in vitro approaches base on the PSC differentiation within embryoid bodies (EBs) and EB outgrowths (EBOs). In such three-dimensional structures molecular and cellular events occurring during the early stages of embryonic development are recapitulated (e.g., [10,11,12]). Culturing EBs and EBOs under defined conditions could lead to the formation of myogenic precursor cells, myoblasts, and myotubes (e.g., [13,14,15]). Until now none of these methods allowed to study the terminal stages of myogenesis, i.e., formation of innervated myofibers. However, promising results, have been presented by Chal et al. and by Mazaleyrat et al. who used two-dimensional cell culture [7,16]. The other model that could be used to analyze advanced stages of differentiation are teratomas, i.e., non-malignant tumors formed from differentiating PSCs. Thus, PSCs injected into the intracapsular space of kidneys or under the skin of mice differentiate spontaneously into the cells and tissues originating from three germ layers. Many studies validated this model as a tool to test pluripotency of mouse [17,18] or human cells [19,20,21], including first iPSCs derived (e.g., [22,23]). It was also applied to study genetically modified PSCs (e.g., [24,25]).

Among the factors which role in myogenesis was extensively studied is PAX7. In mice, ablation of this factor leads to early postnatal lethality [26]. Lack of the functional PAX7, however, does not prevent myogenesis but affects its progression ([4,25,27,28]). Importantly, in *Pax7*-null mice the number of muscle-specific stem cells, i.e., satellite cells (SCs), is lower in skeletal muscles [29,30] and tunica muscularis of the esophagus [31]. Experiments involving selective depletion of *Pax7*-expressing SCs pointed out the differences in the role of this factor in embryonic, early postnatal, and adult myogenesis (reviewed in [32,33]).

In developing embryos myogenic precursor cells (MPCs) originate from somitic mesoderm that cells express *Pax3* and *Pax7* [34,35,36]. During more advanced steps of myogenesis expression of both genes is progressively downregulated and myogenic regulatory factors (MRFs)-MYOD, MYF5, MYOGENIN, and MRF4 are synthesized (e.g., [37,38,39]). Some of the MPCs retain *Pax7* expression, do not differentiate, and become quiescent SCs. Skeletal muscle injury leads to SC activation and differentiation resembling the process of embryonic myogenesis. Many lines of evidence indicate that PAX7 is involved in regulating balance between self-renewal and differentiation of SCs. In differentiating cells PAX7 controls the expression of such factors as MYOD (e.g., [40]). In quiescent SCs it induces expression of inhibitor of differentiation 3 (ID3), which prevents *Myod1* or *Myf5* expression [41]. PAX7 was also shown to be involved in the regulation of proliferation. Analyses of in vitro cultured myoblasts brought contradictory results documenting that *Pax7* overexpression either increased [42] or inhibited proliferation [43]. Our analyses revealed that in the absence of functional PAX7 proliferation of differentiating ESCs increased in vitro [4,14,24] as well as in vivo after transplantation to the mouse muscle [4]. In the latter case, the number of *Pax7*−/− ESCs present within regenerating muscles was significantly increased, as compared to control [4]. Using 5azaC we showed that in the absence of functional PAX7 levels of mRNAs and proteins coding myogenic markers, such as PAX3, MYF5, MYOGENIN, were higher as compared to wild type cells [4]. Surprisingly, PAX7-deficiency had similar impact on the proliferation of mouse embryonic fibroblasts [14]. Moreover, PAX7 was shown to inhibit apoptosis [28] since its absence leads to increased mortality of SCs [34] as well as rhabdomyosarcoma cells [44]. Cell cycle phenotype of *Pax7*−/− ESCs was also suggested by in vivo analyses of teratomas, e.g., in the absence of functional PAX7 teratoma weight increased [25]. However, detailed studies on the cell cycle regulation using teratoma in vivo model was not presented, so far.

Existing data documents that expression of CDK inhibitors is controlled by the cytosine methylation. DNMT3B (DNA cytosine-5-methyltransferase 3 beta) together with DNMT3A catalyzes de novo DNA methylation which is associated with gene silencing [45], including genes encoding cell cycle regulators. In human umbilical cord blood-derived stem cells DMNT3b downregulation increases the levels of CDKIs (e.g., [46,47]). Low levels of DNMTs are associated with the upregulation of p21CIP1 in SCs [48]. On the other hand, DNMT3b together with EZHT2 methyltransferase were shown to be necessary to repress PAX7 during SC differentiation [49]. It was suggested that PAX7 controls the regulation of gene expression via collaboration with APOBEC2 (apolipoprotein B mRNA editing enzyme catalytic polypeptide 2). APOBEC2 is a cytidine deaminase enzyme possibly involved in such processes as RNA editing but also DNA methylation [50]. It is expressed in cardiac and skeletal muscle [51] and was shown to impact myoblast differentiation [50,52,53]. So far, no data has been published concerning PAX7 impact on the cell cycle via modulation of methylation.

To further explore PAX7 function in myogenic differentiation we analyzed two types of pluripotent stem cells, i.e., ESCs and iPSCs derived according to classical protocol involving *Pou5f1*, *Sox2*, *Klf4*, and *c-Myc* [23]. Differentiation of these PSCs was induced in vitro by 5azaC treatment or in vivo within teratomas. Using these models, we studied the interplay between PAX7 and DNMT3b and APOBEC2 known to play a role in the regulation of DNA methylation.

## 2. Materials and Methods

### 2.1. Pluripotent Stem Cell Lines

Embryonic stem cells (ESCs) used in the present study were previously derived and characterized by us [4,14,24,25]. All experiments were carried out on three wild type *Pax7*+/+ ESCs lines (B3, B5, B8) and three knock-out *Pax7*−/− ESCs lines (B4, AI7.15, T2M4). Induced pluripotent stem cells (iPSCs) were obtained from *Pax7*+/+ (W65.5, W65.3, W65.5.1), and *Pax7*−/− (K64.2, K64.3, K64.3.1, K64.6) mouse embryonic fibroblasts by lentiviral transformation conducted and validated by specialized company Stemgent Inc. MA, USA, according to protocol described by Takahasi and Yamanaka [23]. Each experiment and analysis involving these cells was performed in at least three independent replicates.

### 2.2. Preparation of Feeder Cells

Feeder cells, i.e., inactivated mouse embryonic fibroblasts (MEFs), were prepared according to Robertson [54]. Briefly, males and females of F1(C57Bl6NxCBA/H) mice were crossed and 13.5 days after the detection of vaginal plug embryos were dissected to derive primary MEFs. Isolated cells were cultured in DMEM (with 4.500 mg/L glucose, Gibco, Paisley, UK) supplemented with 10% heat-inactivated fetal bovine serum (FBS, Gibco), penicillin and streptomycin (5000 units/mL each, Gibco). After reaching confluency MEFs were inactivated with mitomycin C (10 μg/mL, Sigma-Aldrich, St. Louis, MO, USA), frozen, and seeded.

### 2.3. Genotyping

C57Bl6N females carrying mutation in one allele of Pax7 gene were crossed with 129 Sv males. The 6-week-old F1(C57Bl6Nx129Sv) *Pax7+/−* females were allowed to mate with males of the same cross and genotype. Obtained by crossbreeding mice (tail tips) and isolated as described above, MEFs were genotyped. Briefly, genomic DNA was isolated from MEFs (cells pellets) or tail tips placed in 100 µL of 10% Chelex 100 (Bio-Rad, Hercules, CA, USA) solution in deionized water, in 98 °C, for 15 min. Next, supernatant containing DNA was collected and 1 µL of obtained solution was used for PCR analysis using RedTaq ReadyMix (Sigma-Aldrich) and primers according to conditions described previously [26]. PCR products were separated using 1.5% agarose gel (Bio-Rad) and visualized with ethidium bromide (1 mg/mL, Sigma-Aldrich). Agarose gels were analyzed with GelDoc 2000 (Bio-Rad) using Quantity One software (Bio-Rad). Wild type allele was represented by 200 bp and knock-out allele by 600 bp band [26].

### 2.4. Karyotyping

iPSCs were incubated for 1.5 h in culture medium containing 10 mg/mL of colchicine (Sigma-Aldrich). Next, iPSCs were disaggregated in 0.05% trypsin-EDTA (Invitrogen, Paisley, UK) for 5 min, washed two times in PBS, suspended and incubated for 20 min in 0.56% KCl (Sigma-Aldrich) at room temperature. Cells were fixed with methanol:acetic acid solution (3:1) in 4 °C for 16 h. Finally, iPSCs were dropped onto warm slides, allowed to dry and stained with Giemsa (Merck, Darmstadt, Germany) according to the manufacturer’s protocol. Next, specimens were dehydrated in HistoChoice (Sigma-Aldrich), mounted with VectaMount Mounting Medium (Vector Laboratories, Burlingame, CA, USA) and analyzed using transmitted light microscopy (Axioskop, Zeiss, Oberkochen, Germany). For each iPSC line at least 30 metaphase plates were analyzed.

### 2.5. In Vitro Differentiation of PSCs

PSCs, i.e., ESCs or iPSCs, were cultured as described before [4,24] using so called standard ESC medium composed of KnockOut Dulbecco’s modified Eagle’s medium (KnockOut DMEM, Gibco), 15% high-quality bovine serum (FBS, Gibco), nonessential amino acids (0.1 mM, Gibco), L-glutamine (2 mM, Gibco), β-mercaptoethanol (0.1 mM, Sigma-Aldrich), penicillin and streptomycin (5000 units/mL each, Gibco), murine leukemia inhibitory factor (LIF, 1000 IU/mL, ESGRO, Merck). Before 5-azacytidine (5azaC) incubation, PSCs were separated from MEFs by pre-plating. To this point cell suspension was plated on 1% gelatin coated dishes and incubated in 37 °C, 5% CO_2_, for 20 min. Such procedure was repeated twice allowing MEFs to attach to the dish and ESCs to remain suspended in the medium. After pre-plating PSCs were harvested and 2 × 10^5^ cells were seeded onto 1% gelatin-coated cover slips placed in 35 mm dishes. PSCs were cultured without MEFs in standard ESC medium. After 24 h of culture cells were divided into two groups: control-one and treated with 5azaC (5 µM, Sigma-Aldrich). They were incubated in medium containing DMEM 4.500 mg/L glucose (Gibco), 10% FBS, (Gibco), 10% HS (horse serum, Invitrogen), penicillin and streptomycin 5000 units/mL each (Gibco), containing 5azaC—5 μmol/L (Sigma-Aldrich). After 24 h incubation cells were washed twice with PBS and cells were cultured in the medium lacking 5azaC. Medium was changed every 2 days until the experiment was terminated after 10 days.

### 2.6. In Vivo Differentiation of PSCs–Teratomas Formation

PSCs were cultured under standard conditions to support pluripotency, as described before [4,24]. After 4–5 days of culture cell colonies were disaggregated in 0.05% trypsin/EDTA (Invitrogen) for 3–5 min. Finally, 1 × 10^7^ cells were suspended in 100 µL 0.9% NaCl and injected subcutaneously to isoflurane-anesthetized 3-month-old F1(C57Bl6Nx129Sv) males. Thirty days after transplantation teratomas that reached 1 cm in diameter were isolated, weighed, frozen in liquid nitrogen cooled isopentane, and stored at −80 °C. Small and poorly differentiated teratomas (diameter ≤ 5 mm), were excluded from the analysis.

### 2.7. Histological Analysis

The 10 μm thick sections were collected from frozen teratomas using a cryostat (Microm HM 505 N; Microm International GmbH, Dreieich, Hessen, Germany), air-dried, stained for 10 min with Harris’s hematoxylin (Sigma-Aldrich) and 40 min with Gomori trichrome (Sigma-Aldrich). Finally, sections were mounted in aqueous permanent mounting medium (Dako, Carpinteria, CA, USA). Pictures were taken using a Nikon TE200 microscope (Nikon Instruments, Tokyo, Japan) and NIS Elements software.

### 2.8. Muscle Isolation

*Gastrocnemius* muscles were isolated from 14 days old F1(C57BI6N x 129Sv) *Pax7*+/+ or *Pax7*−/− males. Immediately after isolation muscles were frozen in liquid nitrogen cooled isopentane for mRNA isolation and preserved at −80 °C. Muscles were isolated from at least three animals per genotype.

### 2.9. RNA Isolation, RT-PCR and qPCR

Total RNA was isolated using mirVana miRNA Isolation Kit (Life Technologies, Carlsbad, CA, USA) from undifferentiated PSCs, 5azaC-treated cells, teratomas, as well as skeletal muscles. For RT-PCR 0.2 μg of RNA was used and the reaction was carried using Titan One Tube RT-PCR System (Roche, Basel, Switzerland) and primers according to conditions previously used by us. PCR products were separated in 1.5% agarose gel and analyzed. Reverse transcription was performed using 1 μg total RNA and RevertAid First Strand cDNA Synthesis Kit (Thermo Fisher Scientific, Waltham, MA, USA) according to manufacturer’s instruction. qPCR was performed using specific TaqMan^®^ probes: Mm02019550_s1 (Nanog), Mm00658129_gH (*Pou5f1*), Mm00516104_m1 (*Klf4*), Mm00432359_m1 (*Ccnd1*), Mm00438070_m1 (*Ccnd2*), Mm01612362_m1 (*Ccnd3*), Mm00438066_m1 (*Ccna2*), Mm00432367_m1 (*Ccne1*), Mm00494449_m1 (*Cdkn2a*), Mm04205640_g1 (*Cdkn1a*), Mm00438168_m1 (*Cdkn1b*), Mm01240113_m1 (*Dnmt3b*), Mm00477588_m1 (*Apobec2*), Mm00435125_m1 (*Myf5*), Mm00440387_m1 (*Myod1*), Mm00446194_m1 (*Myog*), Mm00483191 m1 (*Cdh15*), Mm00488527 m1 (*Sdc4*), Mm01205647_g1 (*Actb*), the TaqMan Gene Expression Master Mix (Life Technologies) and LightCycler 96 instrument (Roche). Data was normalized against *Actb*. Data was also standardized against expression observed in mouse embryos at day 13.5. Amplification curves were analyzed using LightCycler 96 SW1.1 software (Roche) for determination of Ct. 2^−ΔΔCT^ analysis was performed according to Livak and Schmittgen [55].

### 2.10. Immunolocalization

Cell cultures or teratoma cryosections were fixed with 3% paraformaldehyde (Sigma-Aldrich) in PBS, at room temperature, for 10 min. Then permeabilization was performed with 0.05% Triton-X 100 (Sigma-Aldrich) in PBS, at room temperature for 3 min. Nonspecific antibody binding sites were blocked by the incubation in 3% bovine serum albumin (BSA, Sigma-Aldrich), at room temperature, for 30 min. Next, specimens were incubated in primary antibodies solutions, i.e., against Ki67 (AB15580, 1:500, Abcam, Cambridge, UK), cleaved Caspase-3 (Asp175, 9661s, 1:200, Cell Signaling, Danvers, MA, USA), OCT3/4 (sc 5279, 1:100, Santa Cruz Biotechnology, Santa Cruz, CA, USA), or NANOG (RCAB0002P-F, 1:50, Cosmo Bio Co., Tokyo, Japan) diluted in 0.5% BSA in PBS, at 4 °C, overnight. Afterwards, specimens were incubated with appropriate secondary antibody conjugated with Alexa 488 (Life Technologies) or Alexa 594 (Life Technologies) diluted 1:200 in 0.5% BSA in PBS, at room temperature, for 2 h. DRAQ5 (Biostatus Limited, Loughborough, UK) diluted 1:500 in PBS were used to visualize the nuclei. Finally, specimens were mounted with Fluorescent Mounting Medium (DakoCytomation, Glostrup, Denmark). The specificity of primary antibodies was verified by incubating samples with secondary antibodies only. The specimens were analyzed using Axio Observer Z1 scanning confocal microscope (Zeiss) equipped with LSM 700 software (Zeiss).

### 2.11. Global DNA Methylation Measurement

Isolation with Wizard^®^ Genomic DNA Purification Kit (Promega, Madison, Wisconsin, USA) was performed to obtain genomic DNA from frozen ESC teratomas. Then, global DNA methylation was quantified using MethylFlash Global DNA Methylation (5-mC) Kit (Epigentek, Farmingdale, NY, USA), according to the manufacturer’s instructions. In brief, 5-methyl cytosine was detected using an ELISA-like reaction. Levels of 5-methyl cytosine in DNA of all biological samples were reported as the amount of methylated cytosines relative to the genomic cytosine content (%). Fluorometric assays were conducted according to the manufacturer’s instructions using 100 ng of input genomic DNA. All samples contained the same amount of DNA. Absorbance was calculated using a microplate reader (Biotek ELx800, Bad Friedrichshall, Germany) at 450 nm. Absolute amounts and the proportion of 5-methyl cytosine were estimated using a standard curve Global measurement of DNA methylation.

### 2.12. Data Analysis

Sample size was computed based on GPower 3.1.9.4 (Informer Technologies, Inc, Los Angeles, CA, USA) to ensure adequate power of the test. Data was analyzed and visualized using Prism version 7.0 (GraphPad Software, Inc. San Diego, CA, USA). All analyses were performed at least in three independent experiments. At first, Shapiro–Wilk test was used to test the distribution of the data. Next, Fisher’s F test was applied to compare the respective variances of the two groups. When the distribution of data was normal, we used Student’s unpaired *t*-test (two-tailed) for comparisons between two groups. If data was characterized by non-Gaussian distribution, we performed Mann–Whitney test. Values of *p* < 0.05 were considered statistically significant (*p* < 0.05 (*), *p* < 0.01 (**), *p* < 0.001 (***) and *p* < 0.0001 (****)). Two-way ANOVA with Sidak’s multiple comparisons or Kruskal–Wallis tests were used to analyze differences between various groups. The level of significance was set at *p* < 0.05.

## 3. Results

### 3.1. Proliferative Status of Cells Building Pax7+/+ and Pax7−/− Teratomas

**Figure 1:**
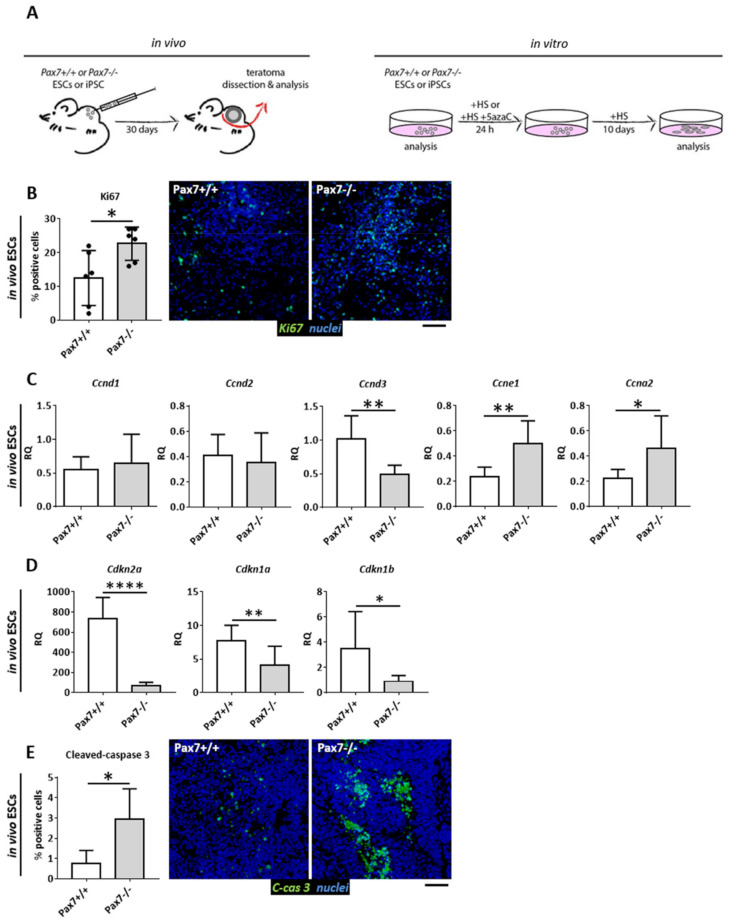
Cell proliferation and apoptosis in the teratomas generated from *Pax7*+/+ and *Pax7*−/− ESCs. (**A**) Experimental design. (**B**) Proportion of Ki67 positive (Ki67+) cells and immunolocalization of Ki67 (green) and nuclei (blue). Scale bar 100 µm. (**C**) Expression of mRNAs encoding cyclin D1 (*Ccnd1*), cyclin D2 (*Ccnd2*), cyclin D3 (*Ccnd3*), cyclin E1 (*Ccne1*), cyclin A2 (*Ccna2*). (**D**) Expression of mRNAs encoding p16INK4A (*Cdkn2a*), p21CIP1 (*Cdkn1a*), p27KIP1 (*Cdkn1b*). (**E**) Proportion of cleaved-caspase 3 (C-cas 3) positive cells and immunolocalization of cleaved-caspase 3 (green) and nuclei (blue). Scale bar 100 µm. White bars—values for *Pax7*+/+ teratomas; gray bars—values for *Pax7*−/− teratomas. Expression was related to the levels assessed in 13.5 d.p.c. mouse embryo (E13.5) and normalized to mRNA encoding β-actin (*Actb*). For each experimental group the *n* ≥ 3. Data are presented as mean ± SD. Stars represent results of Student’s unpaired two-tailed *t*-test: * *p* < 0.05, ** *p* < 0.01, **** *p* < 0.001.

In the current study we decided to follow the cell cycle regulation in PSCs stimulated to differentiate in vivo in teratomas and in vitro by the use of 5azaC, an agent inhibiting DNA methyltransferases. We analyzed wild-type (*Pax7*+/+) and Pax7-null (*Pax7*−*ax*) mouse ESCs and iPSCs. iPSCs were generated from *Pax7*+/+ and *Pax7*−*ax* mouse embryonic fibroblasts by *Pou5f1*, *Sox2*, *Klf4*, and *c*-*Myc* overexpression [23]. ESCs or iPSCs were injected under the skin of mice of the same genetic background and teratomas were isolated 30 days later (Figure 1A). Each presented result came from analyses of three independent ESC and three independent iPSC lines—*Pax7*+/+ (*n* = 3) and *Pax7*−*ax* (*n* = 3). ESC lines used by us were previously characterized, proven to be pluripotent and able to differentiate into ecto-, endo-, and mesoderm derived tissues [24]. iPSC lines were also analyzed by us (Figure S1). In in vitro culture they formed typical PSC colonies (Figure S1A) and their karyotype was normal (Figure S1B). They also synthetized OCT3/4 and NANOG (Figure S1C). Finally, teratomas derived from them contained tissues of ecto-, endo-, and mesodermal origin (Figure S1D). Thus, similarly to ESCs, they fulfilled the criteria of pluripotent stem cells.

First, we analyzed ESC-derived teratomas. The proportion of cycling cells was assessed using the immunodetection of Ki67, i.e., a marker of proliferating cells [56]. Analysis of teratoma sections revealed significantly higher number of proliferating cells in the absence of functional PAX7 (Figure 1B). At the same time, we did not observe any differences in the levels of transcripts encoding G1 phase regulators, i.e., cyclin D1 and D2. Significantly lower level of cyclin D3 mRNA was detected in *Pax7*−*ax* tissues, as compared to control ones (Figure 1C). Next, in the absence of functional PAX7 expression of mRNAs encoding cyclin E1 and A2 was upregulated (Figure 1C). At the same time, the levels of transcripts encoding CDK inhibitors p16INK4A, p21CIP1, and p27KIP1 were significantly lower (Figure 1D). Lack of antiapoptotic action of PAX7 and lower level of p27KIP1 [57] resulted in the increased number of cells in which caspase 3 was activated (Figure 1E).

**Figure 2:**
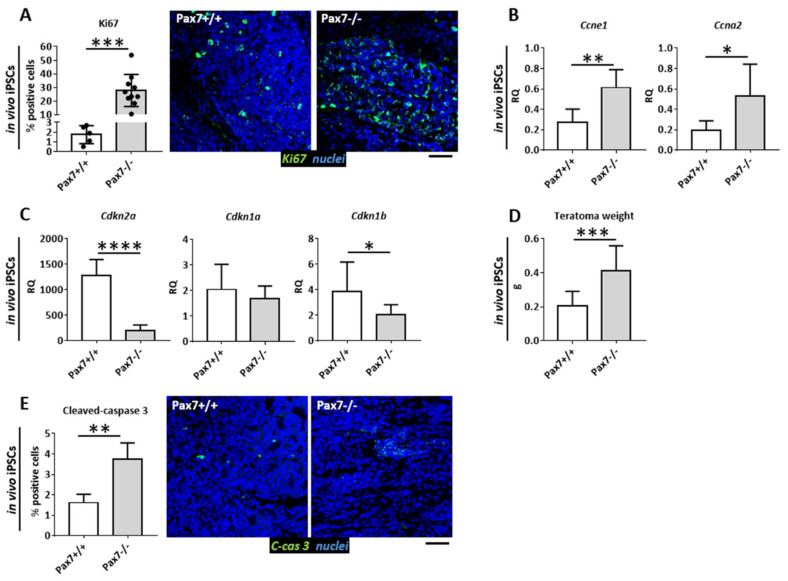
Cell proliferation and apoptosis in the teratomas generated from *Pax7*+/+ and *Pax7*−/− iPSCs. (**A**) Proportion of Ki67 positive (Ki67+) cells and immunolocalization of Ki67 (green) and nuclei (blue). Scale bar 100 µm. (**B**) Expression of mRNAs encoding cyclin E1 (*Ccne1*), cyclin A2 (*Ccna2*). (**C**) Expression of mRNAs encoding p16INK4A (*Cdkn2a*), p21CIP1 (*Cdkn1a*), p27KIP1 (*Cdkn1b*). (**D**) Weight of teratomas. (**E**) Proportion of cleaved-caspase 3 (C-cas 3) positive cells and immunolocalization of cleaved-caspase 3 (green) and nuclei (blue). Scale bar 100 µm. White bars—values for *Pax7*+/+ teratomas; gray bars—values for *Pax7*−/− teratomas. Expression was related to the levels observed in 13.5 d.p.c. mouse embryo (E13.5) and normalized to mRNA encoding β-actin (*Actb*). Data are presented as mean ± SD. Stars symbolizes results of Student’s unpaired two-tailed *t*-test: * *p* < 0.05; ** *p* < 0.01, *** *p* < 0.001, **** *p* < 0.0001.

Teratomas formed from iPSCs mimicked the phenotype of ESC-derived ones. Proliferation (Figure 2A) and cyclin E1 and A2 encoding mRNA expression was increased, as compared to control (Figure 2B). p16INK4a and p27KIP1 encoding mRNAs were downregulated (Figure 2C). Moreover, the weight of *Pax7*−*ax* iPSC derived teratomas was higher (Figure 2D) what was in agreement with previously published data on ESC derived teratomas [25]. Again, lack of antiapoptotic action of PAX7 resulted in the increased number of cells in which caspase 3 was activated (Figure 2E).

### 3.2. Relation between DNA Methylation, PAX7, and Cell Cycle Regulation in In Vitro and In Vivo Differentiating Pax7+/+ and Pax7−/− PSCs

**Figure 3:**
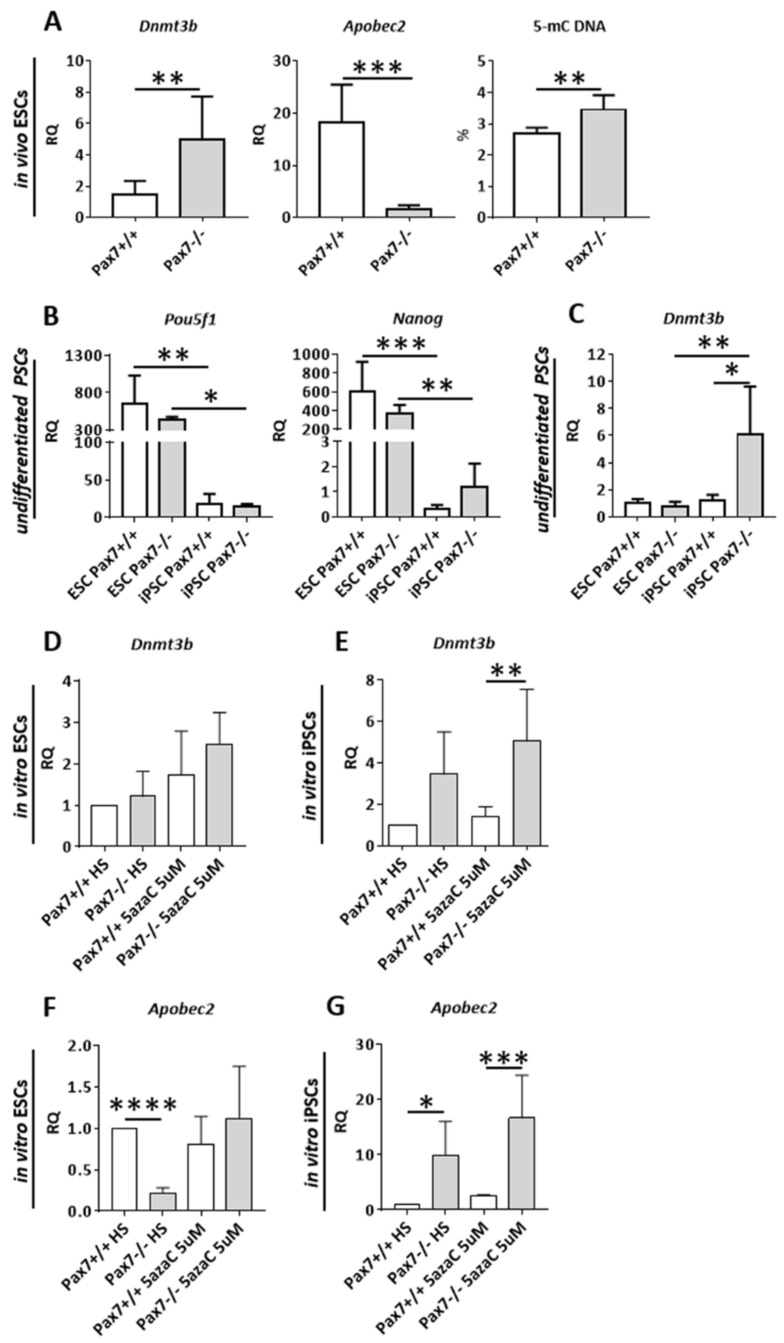
Analysis of factors regulating pluripotency and DNA methylation in *Pax7*+/+ and *Pax7*−/− ESC and iPSCs—undifferentiated and differentiating in vitro treated with HS and 5-azacytidine and in vivo in teratomas. (**A**) Expression of mRNAs *Dnmt3b* and *Apobec2* in the teratomas generated from *Pax7*+/+ and *Pax7*−/− ESCs. Global DNA methylation (5-mC, proportion of methylated cytosines relative to the cytosine genomic content). (**B**) Expression of mRNAs encoding OCT3/4 (*Pou5f1*) and NANOG (*Nanog*) in undifferentiated *Pax7*+/+ and *Pax7*−/− ESCs and iPSCs. (**C**) Expression of mRNAs encoding DNMT3b (*Dmt3b*) in undifferentiated *Pax7*+/+ and *Pax7*−/− ESCs and iPSCs. (**D**) Expression of mRNA encoding *Dnmt3b* in differentiating *Pax7*+/+ and *Pax7*−/− ESCs treated with HS and 5azaC. (**E**) Expression of mRNAs encoding *Dnmt3b* in differentiating *Pax7*+/+ and *Pax7*−/− iPSCs treated with HS and 5azaC. (**F**) Expression of mRNAs encoding *Apobec2* in differentiating *Pax7*+/+ and *Pax7*−/− ESCs treated with HS and 5azaC. (**G**) Expression of mRNAs encoding *Apobec2* in differentiating *Pax7*+/+ and *Pax7*−/− iPSCs treated with HS and 5azaC. White bars—values for *Pax7*+/+ teratomas; gray bars—values for *Pax7*−/− teratomas. Expression was related to the levels observed in 13.5 d.p.c. mouse embryo (E13.5) and normalized to mRNA encoding β-actin (*Actb*). Data are presented as mean ± SD. (A) Stars represent results of Student’s unpaired two-tailed *t*-test: ** *p* < 0.01, *** *p* < 0.001. (B-*Klf4*) Data characterized by non-normal distribution—stars symbolize results of Kruskal–Wallis and post-hoc Dunn’s multiple comparisons test. The level of significance was set at *p* < 0.05. (B-*Pou5f1*, *Nanog, Dnmt3b*, D–G) Stars symbolize result of two-way ANOVA and post-hoc Sidak’s multiple comparisons test: * *p* < 0.05; ** *p* < 0.01, *** *p* < 0.001, **** *p* < 0.0001.

As previously shown, the expression of CDK inhibitors is controlled by the cytosine methylation driven by DNMTs (e.g., [46,47,48]). APOBEC2, on the other hand, is involved in the regulation of myoblast differentiation [50,52,53]. To look into the relation between PAX7, DNA methylation, and cell cycle regulation we first analyzed ESC derived teratomas. We found out that in the absence of functional PAX7 *Dnmt3b* expression was significantly upregulated (Figure 3A). We observed similar increase in iPSC-derived *Pax7*−*ax* teratomas (data not shown). At the same time dramatic drop in the levels of *Apobec2* mRNA and a significant increase in the overall level of 5-methyl cytosine in DNA were observed (Figure 3A). Again, *Apobec2* downregulation was observed in iPSC-derived *Pax7*−*ax* teratomas (data not shown).

Thus, increase in *Dnmt3b* expression led to downregulation of CDKIs expression, what was observed both in ESC- and iPSC-derived *Pax7*−*ax* teratomas (Figure 1D and Figure 2C). This could prevent efficient cell cycle arrest and cell differentiation. However, increase in *Apobec2* expression could facilitate the upregulation of MRFs leading to myogenic differentiation. Such phenomenon was previously described using the same experimental model [25].

Next, we analyzed in vitro cultured ESCs and iPSCs. Analysis of two pluripotency makers *Pou5f1* and *Nanog* expression showed that it was significantly higher in pluripotent ESCs than in iPSCs (Figure 3B). The level of *Pou5f1* and *Nanog* mRNAs did not depend on genotype and was comparable in each type of cell line analyzed. iPSCs were also characterized by the lower levels of *Dnmt3b* mRNA, as compared to ESCs. Interestingly, *Dnmt3b* expression was upregulated in iPSCs lacking functional PAX7 (Figure 3C).

To have a closer look at the PAX7 and methylation interplay we induced PSCs to differentiate using horse serum (HS) and 5azaC. 5azaC decreases the level of cytosine methylation. In case of PSCs it induces OCT3/4 and NANOG degradation mediated by caspase 3 and 7 [58]. Importantly, 5azaC was also shown to promote myogenic differentiation and induce MYOD1 expression in human [59] and mouse ESCs [6]. *Pax7*+/+ and *Pax7*−*ax* ESCs and iPSCs were cultured in medium supplemented with HS and 5 µM 5azaC for 24 h and then in medium containing HS for 10 days. Control cells were cultured in the medium lacking 5azaC (Figure 1A). Under such experimental conditions, the morphology of the cells changed (Figure S2A). *Dnmt3b* expression was elevated only in differentiating *Pax7*−*ax* iPSCs (Figure 3D,E), i.e., the cells with initially low *Pou5f1* and *Nanog*, as compared to ESCs. 5azaC led to the upregulation of *Apobec2* expression, again only in iPSCs that lacked functional PAX7 (Figure 3F,G). Interestingly, *Pax7*−*ax* ESCs cultured only in the presence of horse serum [HS, (Figure 3F,G)] showed downregulation of *Apobec2*, similarly as observed in ESC-derived *Pax7*−*ax* teratomas (Figure 3A). In case of iPSCs-cultured in HS *Apobec2* was upregulated (Figure 3G).

### 3.3. Proliferation of Pax7+/+ and Pax7−/− Differentiating PSC

**Figure 4:**
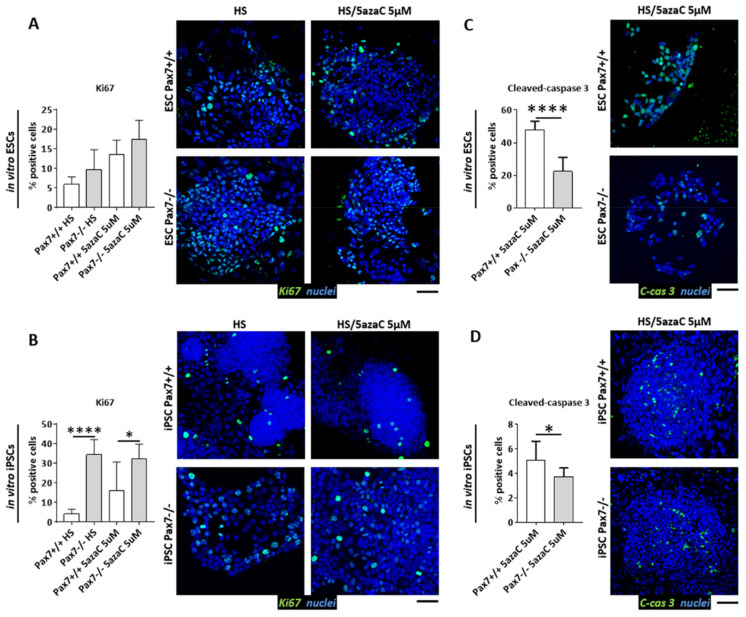
Cell proliferation and apoptosis in differentiating *Pax7*+/+ and *Pax7*−/− ESCs and iPSCs treated with HS and 5-azacytidine. (**A**) Proportion of Ki67 positive (Ki67+) cells and immunolocalization of Ki67 (green) and nuclei (blue) in ESCs. Scale bar 100 µm. (**B**) Proportion of Ki67 positive (Ki67+) cells and immunolocalization of Ki67 (green) and nuclei (blue) in iPSCs. Scale bar 100 µm. (**C**) Proportion of cleaved-caspase 3 (C-cas 3) positive cells and immunolocalization of cleaved-caspase 3 (green) and nuclei (blue) in ESCs. Scale bar 100 µm. (**D**) Percentage of cleaved-caspase 3 (C-cas 3) positive cells and immunolocalization of cleaved-caspase 3 (green) and nuclei (blue) in iPSCs. Scale bar 100 µm. White bars—values for *Pax7*+/+ PSCs; gray bars—values for *Pax7*−/− PSCs. Data are presented as mean ± SD. (**A**,**B**) Stars symbolize result of two-way ANOVA and post-hoc Sidak’s multiple comparisons test: * *p* < 0.05, **** *p* < 0.0001. (**C**,**D**) Stars symbolize results of Student’s unpaired two-tailed *t*-test: * *p* < 0.05, **** *p* < 0.0001.

**Figure 5:**
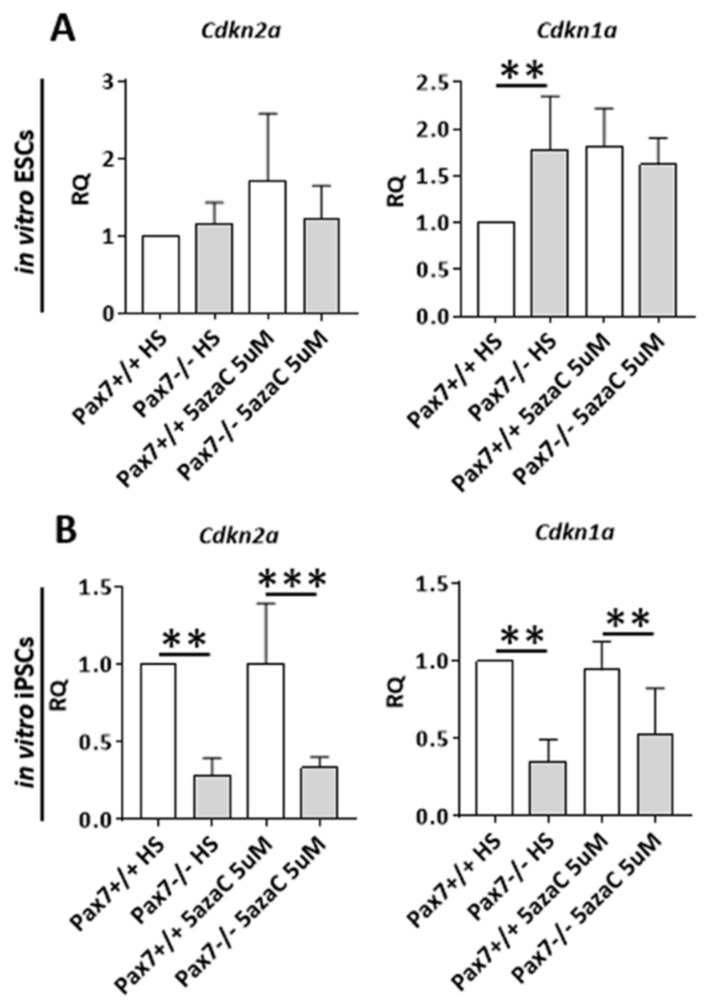
Cell cycle inhibitors in differentiating *Pax7*+/+ and *Pax7*−/− ESCs and iPSCs treated with HS and 5-azacytidine. (**A**) Expression of mRNAs encoding p16INK4A (*Cdkn2a*), p21CIP1 (*Cdkn1a*) in differentiating Pax7+/+ and Pax7−/− ESCs treated with HS and 5azaC. (**B**) Expression of mRNAs encoding p16INK4A (*Cdkn2a*), p21CIP1 (*Cdkn1a*) in differentiating Pax7+/+ and Pax7−/− iPSCs treated with HS and 5azaC. White bars—values for *Pax7*+/+ ESCs and iPSCs; gray bars—values for *Pax7*−/− ESCs and iPSCs. Expression was related to the levels observed in 13.5 d.p.c. mouse embryo (E13.5) and normalized to mRNA encoding β-actin (*Actb*). Data are presented as mean ± SD. Stars symbolize result of two-way ANOVA and post-hoc Sidak’s multiple comparisons test: ** *p* < 0.01, *** *p* < 0.001. Scale bar 100 µm.

Since differentiating *Pax7*−/− ESC and iPSC lines analyzed by us differed in the levels of *Dnmt3b* and *Apobec2* expression we asked how such phenotype impacts the cell cycle. To elucidate this issue, we used ESCs and iPSCs induced to differentiate by HS and 5µM 5azaC treatment, as described above (Figure 1A). The absence of functional PAX7 did not impact the number of Ki67+ positive ESCs (Figure 4A). However, cells expressing this marker were significantly more abundant in cultures treated with HS and 10µM 5azaC (data not shown). In case of *Pax7*−/− iPSC cultures, the number of proliferating cells was considerably increased in every group studied (Figure 4B). Moreover, the number of *Pax7*−/− ESCs as well as iPSCs with activated caspase 3 was lower, as compared to wild type controls (Figure 4C,D). In in vitro differentiating ESCs, 5azaC did not impact the levels of *Cdkn2a* and *Cdkn1a*, encoding p16INK4a or p21CIP1 inhibitors, regardless of their genotype (Figure 5A).

The levels of abovementioned RNAs were significantly lower in *Pax7*−/− iPSCs (Figure 5B). Thus, the comparison of in vitro cultured ESCs and iPSCs uncovered the relationship between PAX7 and methylation regulation. In the absence of PAX7, differentiating iPSCs significantly increased *Dnmt3b* expression. *Cdkn2a* and *Cdkn1a* mRNAs and number of proliferating cells were increased (Figure 4B and Figure 5B). *Apobec2* upregulation observed by us in *Pax7*−/− iPSCs led to increase in the *Myog* expression (Figure S2B).

### 3.4. Dnmt3a, Apobec2, and CDKIs in Pax7+/+ and Pax7−/− Skeletal Muscles

To verify PAX7 impact at the DNA methylation in vivo we assessed the levels of mRNAs encoding APOBEC2, DNMT3B, CDKIs, and SC markers (MYF5, M-cadherin, syndecan 4) in *Gastrocnemius* muscles of two-week old *Pax7*+/+ and *Pax7*−/− mice. *Apobec2* expression was significantly downregulated while increase in the level of *Dnmt3b* was insignificant (*p* = 0.08) in *Pax7*−/− muscles (Figure S3A). Levels of mRNAs encoding p21CIP1 and p27KIP1 were also decreased (Figure S3B). Thus, “muscle phenotype” reflected the one of *Pax7*−/− teratomas. Finally, *Myf5*, *Cdh15* (M-cadherin), and *Sdc4* (syndecan 4) mRNA levels were significantly lower in *Pax7*−/− muscles, as compared to control (Figure S3C). Thus, it was in agreement with the previous reports showing the lower number of SCs in *Pax7*-null skeletal muscles [29,30] and also in teratomas derived from *Pax7*-deficient PSCs [25].

Summarizing, we documented that PAX7 controls proliferation/differentiation balance by blocking the expression of *Dnmt3b* what leads to the upregulation of CDKIs. Next, it positively influences APOBEC2 leading to the demethylation of sequences regulating MRF genes what promotes myogenic differentiation.

## 4. Discussion

In the current study, using differentiating PSCs, we focused at the relation between PAX7, factors involved in DNA methylation, and cell cycle progression. We used two different models of PSC differentiation. In vivo generated teratomas and PSCs in vitro induced to differentiate using horse serum and demethylating agent-5azaC. Each of these models was used in the past proving their usefulness for studying PSC phenotypes caused by deficiency of PAX7 function [4,25]. Moreover, our previous studies documented that *Pax7*−/− ESCs were more prone to undergo myogenic differentiation induced in vitro in EBs [24] or by HS and 5azaC [4]. It was manifested by increased expression of myogenesis regulating factors, such as *Pax3*, *Myf5*, *Myod1*. In in vivo developing *Pax7*−/− teratomas, however, the levels of *Myod1* and *Myog* were significantly lower, as compared to wild type control. As a result, myogenic differentiation was delayed [25].

PAX7 is a master regulator of embryonic myogenesis and SC function in the adult muscle. It controls the expression of genes promoting SC survival and self-renewal, and preventing their differentiation (reviewed in [60,61]). Many lines of evidence suggest the involvement of PAX7 in cell cycle control. Thus, it has been shown that myoblasts lacking *Pax7* decreased their proliferation [34] and formed fewer and smaller myotubes [30]. Mice expressing nonfunctional PAX7 were characterized by small myofiber diameter and muscle mass [26,29]. However, it has to be remembered that decrease in PAX7 level accompanies SC activation followed by intensive proliferation and formation of myotubes and myofibers (for review see [60]). Results of our studies focusing at PSCs lacking functional PAX7 documented that indeed, as it happens in the case of SCs, the absence of this transcription factor facilitates the cell cycle progression. Regardless of the method used to induce PSC myogenic differentiation we always observed that dysfunction of PAX7 leads to the increased proliferative potential. In the case of ESCs it was proven using various approaches. In in vitro cultured EBs, by assessing the number of cells and their proliferation rate, detection of Ki67, and mRNAs encoding cell cycle regulators [14]. In vivo by localizing Ki67 expressing ESCs transplanted into regenerating skeletal muscle [4], or by measuring teratoma weight (current study, [25]). Current results confirmed that expression of mRNAs encoding cyclin E1 and A2 was increased and those coding CDKI, i.e., p16IN4A, p21CIP1, and p27KIP1 was significantly reduced in *Pax7*−*ax* ESC and iPSC-derived teratomas. As a result, the number of Ki67 expressing cells was increased. Thus, we documented that PAX7 cell cycle limiting function is the same in SCs and in differentiating PSCs. We also showed slight increase in *Dnmt3b* mRNA level in *Pax7*−*ax* skeletal muscles what was accompanied by the drop in CDKIs expression. The question was how such PAX7 effect is executed.

Several lines of evidence suggested the connection between PAX7 and DNMT3b. DNMT3b, together with EZHT2 methyltransferase, was shown to repress PAX7 and NOTCH1 during SC differentiation [49]. Knockdown of *Dnmt3b* led to the upregulation of *Pax7* expression in early neural crest cells [62]. In the case of human rhabdomyosarcoma cells, it resulted in increase of *MYOD1*, *MYOG*, and *MyHC* expression [47]. Importantly, it was documented that in in vitro differentiating mouse ESCs the level of mRNA encoding *Dnmt3b* changed independently of *Pax7* expression [50]. In the current study, for the first time, we documented closer relation between these two factors. In the absence of functional PAX7 *Dnmt3b* expression increased in ESC- and iPSC-derived teratomas, as well as in vitro differentiating iPSCs. Such increase led to the increase in DNA methylation. Thus, it suggested that PAX7 negatively regulates *Dnmt3b* expression. Such methylation increase led to the drop in the CDKI expression, what we showed for p16INK4A (*Cdkn2a*), p21CIP1 (*Cdkn1a*), and p27KIP1 (*Cdkn1b*). DNMT3b was shown to be responsible for CDKI levels in human umbilical cord blood-derived stem cells [47], human rhabdomyosarcoma cells [48], and many cancer cell lines [63,64,65]. Low levels of DNMT3b in PRMT7 (protein arginine methyl transferase 7) deficient SCs was accompanied by p21CIP1 increase [48]. As far as cell cycle regulation is concerned we also detected cyclin E1 as well as A2 upregulation in *Pax7*−/− PSCs (current study, [14]). In the current study we also show that *Pax7*−/− ESC-derived teratomas express lower levels of cyclin D3. This particular cyclin D is involved in early activation of myogenesis by the increase of *Cdkn1a*, *Myod1*, *Myf5*, and *Myog* expression. Thus, its decrease may also promote proliferation and prevent efficient myogenic differentiation (e.g., [66,67,68]). Interestingly, cyclin D3 also promotes *Pax7* expression [67,68].

Another factor we analyzed was APOBEC2 that was suggested to play a role in many processes like tumorigenesis by RNA editing [69] or tissue regeneration [70], and myogenesis progression by DNA demethylation [50]. *Apobec2*-deiffcient mice were characterized by many defects, including a reduction in body mass, mild myopathy, and increased proportion of slow muscle fibers in Soleus muscle [71]. Next, in the absence of APOBEC2 mitochondrial defects were observed in skeletal muscle what led to increased mitophagy and chronic muscle damage [72]. It was also shown that APOBEC2 function is important for *Myog* expression during myoblast differentiation [50]. We show here that *Pax7*−/− teratomas *Apobec2* expression was decreased. Such downregulation could result in lower levels of *Myf5*, *Myod1*, and *Myog* expression [25]. Our current analyses also document that in *Pax7*−/− skeletal muscles significant downregulation in *Apobec2* mRNA accompanies drop in *Myf5* expression. Taken together, *Dnmt3b* upregulation and *Apobec2* downregulation resulted in the prevalence of proliferation over differentiation in *Pax7*−/− teratomas. Interestingly, “cell cycle phenotype” of in vitro differentiating PSCs differed from that characteristic for teratomas.

In our current study we decided to in vitro culture and analyze two types of PSCs, i.e., ESCs which we previously studied [4,14,24,25], and also iPSCs. Our decision was driven also by the fact, that iPSCs differed to some extent from ESC counterparts. This difference was manifested by lower expression of mRNAs encoding pluripotency markers, i.e., OCT3/4 and NANOG in iPSCs. Interestingly, we also revealed that *Dnmt3b* expression was significantly higher in *Pax7*−/− iPSCs. This analysis confirmed that when interpreting the results and drawing the conclusions from PSC analyses, special attention is needed. Many lines of evidence document certain aberrations manifested in PSCs, including epigenetic ones [73,74,75,76]. For this reason, every analysis presented in the current study was performed using three independent cell lines of each type. Moreover, crucial phenotypes of PSCs concerning *Dnmt3b*, *Apobec2*, and CDKIs expression were confirmed in skeletal muscles.

**Figure 6:**
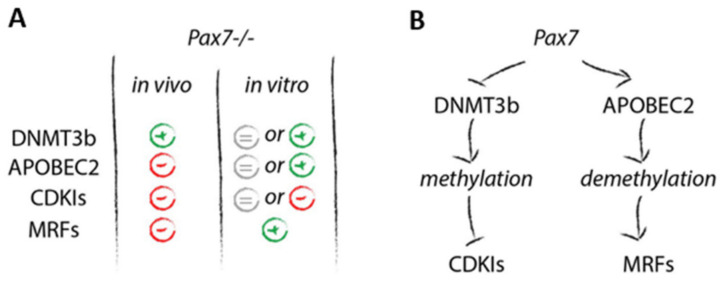
PAX7, DNMT3b, APOBEC2, CDKIs and MRFs in in vitro differentiating ESCs and iPSCs and in vivo developing teratomas. (**A**) Changes in the expression of selected markers in *Pax7*−/− cells in relation to *Pax7*+/+ cells. + higher level; − lower level; = significant difference was not observed. (**B**) Schematic diagram presenting status quo in wild type, i.e., *Pax7*+/+ cells.

Having such tool, we had the opportunity to study PAX7 and methylation at the initially different background. In the case of differentiating ESCs we did not observe any significant changes in *Dnmt3b* mRNA levels, regardless of the genotype. *Apobec2* mRNA, however, was at the lower level in *Pax7*−/− ESCs cultured in the presence of HS only, as compared to wild type control. iPSC response to HS and 5azaC treatment was more evident, as compared to ESCs. In the absence of functional PAX7 we observed *Dnmt3b* and *Apobec2* upregulation accompanied by significant drop in *Cdkn2a* and *Cdkn1a* as well as increase in *Myod1* and *Myog* expression (Figure 6A). Thus, both types of *Pax7*−/− PSCs response to 5azaC treatment with increased proliferation, as shown by the proportion of Ki67+ cells but at the same time differentiation markers were effectively upregulated. Regardless of the model used by us, in vitro or in vivo, PAX7 deficiency resulted in increase of level of *Dnmt3b* followed by decreased level of mRNAs encoding CDKIs. However, *Apobec2* downregulation occurred only in ESCs cultured in vitro in the presence of HS and in vivo in teratomas. As we have previously shown in such cells, MRFs expression and myogenic differentiation were affected [4,25]. When we induced DNA demethylation by 5azaC treatment in in vitro cultured *Pax7*−/− cells we prevented drop in *Apobec2* expression (ESCs) or even led to its increase (iPSCs). Moreover, in *Pax7*−/− skeletal muscles *Apobec2* mRNA level was significantly lower while *Dnmt3b* mRNA was only slightly upregulated. This clearly documented that PAX7 regulates *Dnmt3b* and *Apobec2* expression, and thereby impacts the cell cycle progression.

The question remains what causes the differences between ESCs and iPSCs. First of all, iPSCs analyzed by us were characterized by the lower levels of pluripotency markers. This, however, did not affect their ability to form teratomas containing derivatives of three germ layers, or karyotype. Increased expression of *Dnmt3b* mRNA in *Pax7*−/− iPSCs could be linked with their pluripotency status [77]. Analysis of human iPSC showed that aberrant DNA methylation is associated with the somatic cells reprogramming what can also affect gene expression (for review see [76]). At the end, we obtained differentiating iPSCs, but caution and special attention must be taken, as we mentioned above, to analyze several cell lines of the same genotype.

Apart from the cell cycle regulators, we also compared the apoptosis level in ESCs and iPSC induced to differentiate with 5azaC and teratomas derived from these cells. It allowed us to document significant differences between in vivo and in vitro models of differentiation. In teratomas, in the absence of functional PAX7, we observed significant increase of cells containing active caspase 3. This observation goes with line with the previous data showing antiapoptotic PAX7 function [34,40,78,79]. In vitro setting seems to be more complicated. In the current, as well as in our previous study [14] we observed significantly fewer *Pax7*−/− cells containing activated caspase 3, as compared to wild type control. As we previously suggested, it is possible that in vitro cultured *Pax7*−/− cells might prematurely exit the cell cycle and start to differentiate, what is manifested by an increase in MRFs expression, and CDKIs downregulation. Under such circumstances the apoptosis is not timely executed, as it happens in vivo. Again, this points out the differences between in vivo and in vitro models used by us.

## 5. Conclusions

We proved that in differentiating PSCs PAX7 balances the cell cycle progression via regulating expression of *Dnmt3b* and *Apobec2*. PAX7 blocks the expression of *Dnmt3b* what leads to the upregulation of CDKIs. At the same time, its action restricts *Ccne1* and *Ccna2* expression. It positively influences APOBEC2 leading to the demethylation of sequences regulating MRF genes what induces myogenic differentiation (Figure 6B) [50]. Lack of functional PAX7 results in higher DNA methylation causing delayed cell cycle exit and myogenic differentiation. However, this effect can be neutralized/compensated and even inverted by treatment with demethylating agents.

## Data Availability

The data presented in this study are available on request from the corresponding author.

## Supplementary Materials

The following are available online at https://www.mdpi.com/article/10.3390/cells10092205/s1, Figure S1: Characteristics of *Pax7*+/+ (W65.5, W65.3, W65.5.1) and *Pax7*−/− (K64.2, K64.3, K64.3.1, K64.6) iPSC line. Figure S2: Myogenic differentiation of *Pax7*+/+ and *Pax7*−/− iPSCs treated with HS and 5-azacytidine. Figure S3: Expression of selected genes in muscles obtained from *Pax7*+/+ and *Pax7*−/− 14 day old mice.
