# Supplementary material for: PAX7 Balances the Cell Cycle Progression via Regulating Expression of Dnmt3b and Apobec2 in Differentiating PSCs
Source: Cells. 2021 Aug 26;10(9):2205. doi: 10.3390/cells10092205 (PMC8472244; doi:10.3390/cells10092205)
Supplement: Supplementary file 1 [file cells-10-02205-s001.zip › Figure S1 Florkowska et al R1.pdf]

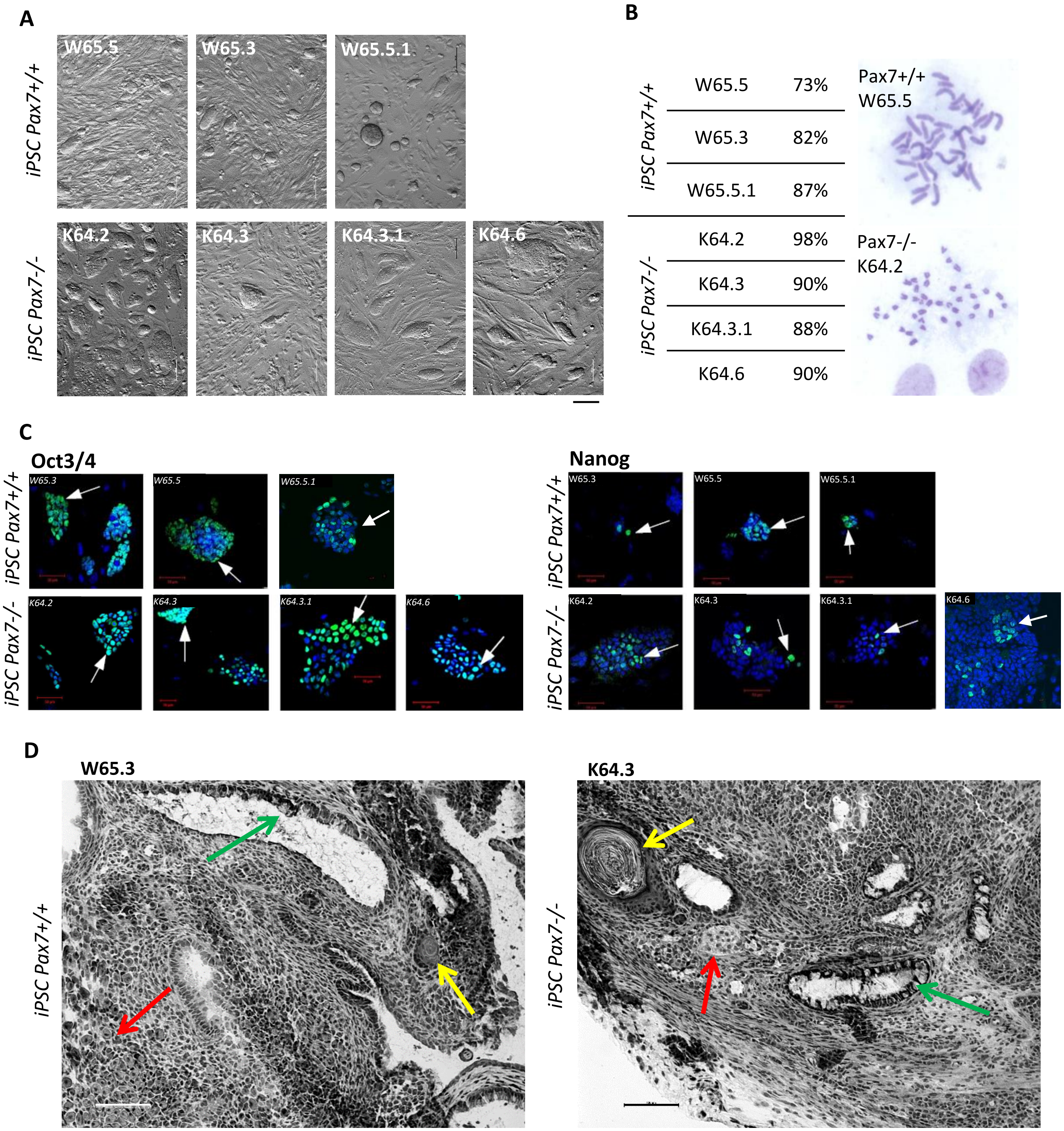

Figure S1. Characteristics of *Pax7*+/+ (W65.5, W65.3, W65.5.1), and *Pax7*-/- (K64.2, K64.3, K64.3.1, K64.6) iPSC line. (A) Morphology of iPSC lines, colonies cultured under pluripotency supporting conditions. Scale bar 100 µm **(B)** Karyotype analysis. Table - proportion of 2n metaphase plates (2n karyotype) in analyzed iPSC lines; at least 30 metaphase plates were analyzed for each cell line. **(C)** Localization of Oct3/4 (green) or Nanog (green) and nuclei (blue) in undifferentiated Pax7+/+ and Pax7-/- iPSCs colonies. Scale bar 50 µm. **(D)** Histological analysis of teratomas derived from Pax7+/+ and Pax7-/- iPSCs; representative photos for Pax7+/+ iPSCs (W65.3) and Pax7-/- iPSC (K.64.3). Sections stained with Harris' Hematoxylin and Gomori's Trichrome. Ectodermal structures – yellow arrows, mesodermal structures –red arrows, endodermal structures - green. Scale bar 100µm.
