# Supplementary material for: PAX7 Balances the Cell Cycle Progression via Regulating Expression of Dnmt3b and Apobec2 in Differentiating PSCs
Source: Cells. 2021 Aug 26;10(9):2205. doi: 10.3390/cells10092205 (PMC8472244; doi:10.3390/cells10092205)
Supplement: Supplementary file 1 [file cells-10-02205-s001.zip › Figure S2 Florkowska et al R1.pdf]

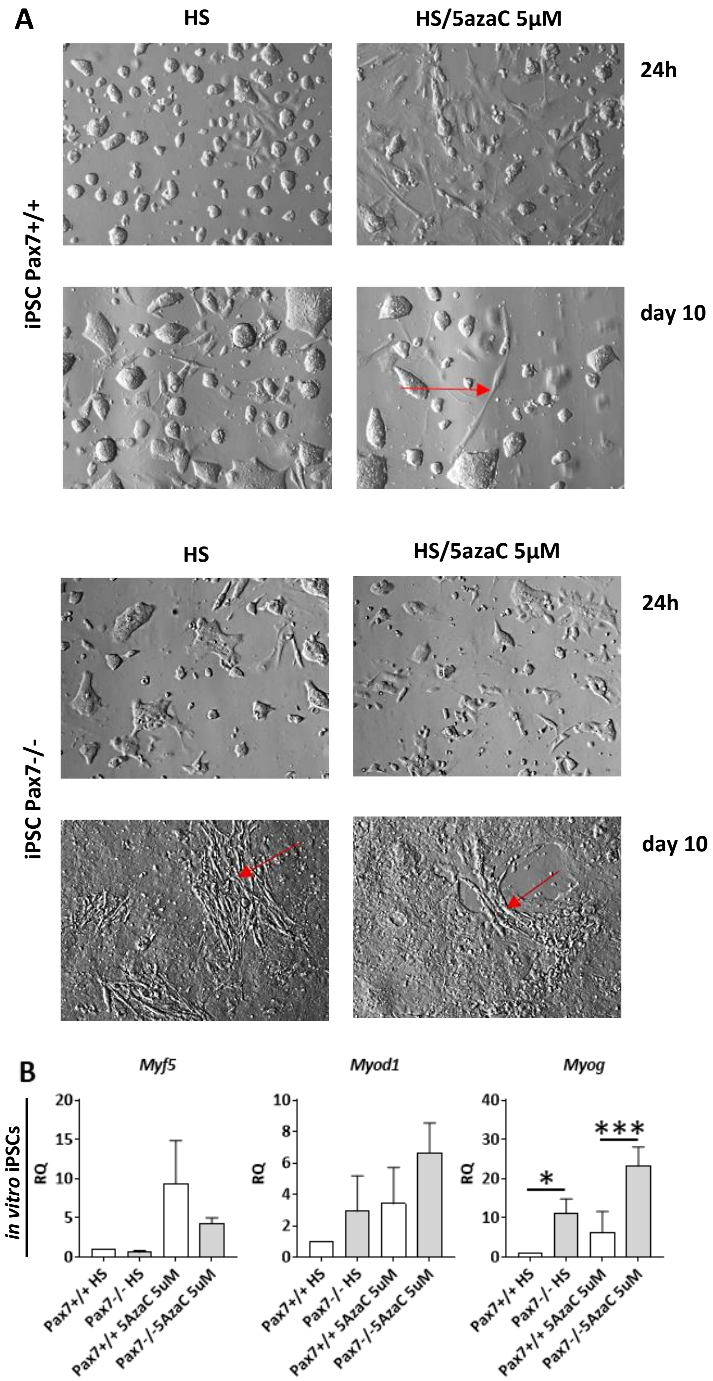

Figure S2. Myogenic differentiation of *Pax7*+/+ and *Pax7*-/- iPSCs treated with HS and 5-azacytidine. (A) Morphology of iPSCs cultured for 24h in medium supplemented with horse serum (HS) and 5µM 5azaC and then for 10 days in medium with HS. Control cells were cultured in the medium lacking 5azaC. Red arrows indicate myotube-like structures. **(B)** Expression of *Myf5*, *Myod1*, and *Myog* mRNAs.

White bars − values for *Pax7*+/+ iPSCs, gray bars − values for *Pax7*-/- iPSCs. Expression was related to the levels observed in 13.5 d.p.c. mouse embryo (E13.5), and normalized to mRNA encoding β-actin (*Actb*). Data are presented as mean±SD. Stars symbolizes result of Two-way ANOVA and post-hoc Sidak's multiple comparisons test: *$p<0.05$; ***$p<0.001$.
