# Supplementary material for: PAX7 Balances the Cell Cycle Progression via Regulating Expression of Dnmt3b and Apobec2 in Differentiating PSCs
Source: Cells. 2021 Aug 26;10(9):2205. doi: 10.3390/cells10092205 (PMC8472244; doi:10.3390/cells10092205)
Supplement: Supplementary file 1 [file cells-10-02205-s001.zip › Figure S3 Florkowska et al R1.pdf]

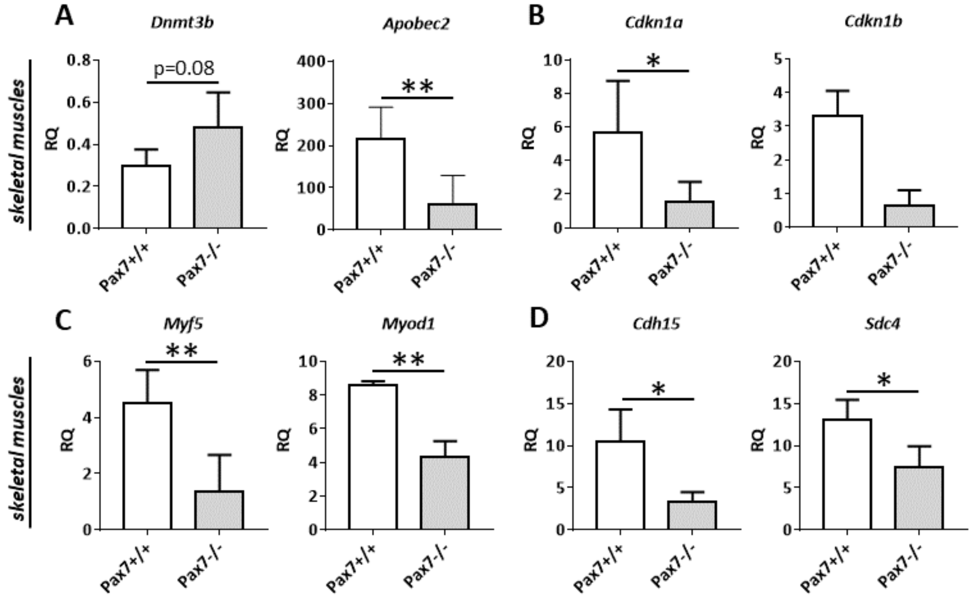

Figure S3. Expression of selected genes in muscles obtained from *Pax7*+/+ and *Pax7*-/- 14 day old mice. (A) Expression of *Dnmt3b* and *Apobec2* mRNAs. **(B)** Expression of *Cdkn1a* and *Cdkn1b* mRNAs. **(C)** Expression of *Myf5* and *Myod1* mRNAs. **(D)** Expression of *Cdh15* and *Sdc4* mRNAs.

White bars – values for *Pax7*+/+ muscles, gray bars – values for *Pax7-/-* muscles. Expression was related to the levels observed in 13.5 d.p.c. mouse embryo (E13.5), and normalized to mRNA encoding β-actin (*Actb*). Data are presented as mean±SD. (A - *Apobec2*) Stars represent results of Student's unpaired two-tailed *t*-test: **p<0.01. (B - *Cdkn1a*) Stars represent results of Student's unpaired two-tailed *t-test*: *p<0.05. (C - *Myf5*) Data characterized by non-normal distribution - stars symbolizes results of Mann Whitney test. The level of significance was set at p<0.05. (C - *Myod1*) Stars represent results of Student's unpaired two-tailed *t*-test: **p<0.01. (D – *Cdh15*, *Sdc4*) Stars represent results of Student's unpaired two-tailed *t*-test: *p<0.05.
